# Complete mitochondrial genome of the summer heath fritillary butterfly, *Mellicta ambigua* (Lepidoptera: Nymphalidae)

**DOI:** 10.1080/23802359.2021.1917318

**Published:** 2021-05-07

**Authors:** Min Jee Kim, Myunghyun Chu, Jeong Sun Park, Sung-Soo Kim, Iksoo Kim

**Affiliations:** a Department of Applied Biology, College of Agriculture & Life Sciences, Chonnam National University, Gwangju, Republic of Korea; b Experiment and Analysis Division, Honam Regional Office, Animal and Plant Quarantine Agency, Gunsan, Republic of Korea; c Research Institute for East Asian Environment and Biology, Seoul, Republic of Korea

**Keywords:** *Mellicta ambigua*, mitochondrial genome, nymphalinae, phylogeny

## Abstract

We sequenced the mitochondrial genome (mitogeome) of the summer heath fritillary bullterfly, *Mellicta ambigua* Ménétriès, 1859 (Lepidoptera: Nymphalidae), which is listed as an endangered insect in South Korea. The 15,205-bp long complete genome contained 13 protein-coding genes (PCGs), 2 rRNA genes, 22 tRNA genes, and 1 A + T-rich region with an arrangement identical to that observed in most insect mitogenomes. Unlike the other PCGs, *COI* had the atypical CGA start codon frequently found in lepidopteran *COI*. The A/T content of the whole mitogenome was 80.57%; however, it varied among the regions/genes as follows: A + T-rich region, 93.39%; *srRNA*, 85.37%; *lrRNA*, 84.92%; tRNAs, 81.13%; and PCGs, 79.22%. Phylogenetic analyses using concatenated sequences of the 13 PCGs and 2 rRNAs placed *M. ambigua* as a sister group to the within-tribe species, *Melitaea cinxia*, with the highest nodal support both in the maximum-likelihood (ML) and Bayesian inference (BI) methods.

The summer heath fritillary, *Mellicta ambigua* Ménétriès, 1859 (Lepidoptera: Nymphalidae), is distributed in Russia, Mongolia, Korea, Japan and northeastern China (Kim and Seo 2012). In Korea, the adults of this species occur once a year, from around June to August, in grasslands located in mountain areas (Nam et al. 1998; Kim 2010). Adults produce several eggs in a mass on the backside of host leaves (e.g. the Culver’s root, *Veronicastrum sibiricum* and the cow wheat, *Melampyrum roseum var. japonicum*), and the fourth instar larvae overwinter as a group in a nest made of silk secreted by the larva and the folded dry leaves of the host plant (Nam et al. 1998). Habitat loss and climate change are the main factors that have placed the species on the endangered list in Korea (Choi and Kim 2012).

One *M. ambigua* adult was collected at Jindo-gun, Jeollanam-do, Korea (34° 22′09″ N, 126° 09′59″ E) in June 2018 after obtaining the necessary permissions (permission no. 2018-23, Yeongsan River Basin Environmental Office, Ministry of Environment, Korea). A voucher specimen was deposited at the National Institute of Biological Resources, Incheon, Korea, with the accession number CNU7298 (Iksoo Kim, ikkim81@chonnam.ac.kr). After DNA was extracted from the hind legs with the Wizard^TM^ Genomic DNA Purification Kit (Promega, Madison, WI, USA), three long overlapping fragments (*COI*-*ND5*, *ND5*-*lrRNA*, and *srRNA*-*COI*) were amplified using three sets of primers designed from the available mitochondrial genomes of Nymphalinae (Hu et al. unpublished, GenBank accession number GQ398377; Chen et al. 2012; McCullagh and Marcus 2015), which were then used as templates for the amplification of 26 short overlapping fragments using the primers reported in Kim et al. (2012). Primer information for long overlapping fragments will be provided upon request. Phylogenetic analyses were performed using 22 mitogenome sequences in the subfamilies Nymphalinae, including *M. ambigua* and Apaturinae. Thirteen protein-coding genes (PCGs) and two rRNA genes were aligned and concatenated (13,153 bp, including gaps). The Maximum-likelihood (ML) and Bayesian inference (BI) methods, implemented in CIPRES Portal v. 3.1 (Miller et al. 2010), were used for phylogenetic analyses. An optimal partitioning scheme and substitution model (GTR + Gamma + I) were determined using PartitionFinder 2 and the Greedy algorithm (Lanfear et al. 2012, 2014, 2016).

The 15,205-base pair (bp)-long complete mitochondrial genome of *M. ambigua* was composed of typical gene sets (2 rRNAs, 22 tRNAs, and 13 PCGs) and a major non-coding A + T-rich region (GenBank acc. no. MK252271). The length of the *M. ambigua* A + T-rich region was 333 bp, which was well within the range found in other sequenced Nymphalinae species (126 bp in *Yoma sabina*, Unpublished, GenBank acc. no. KF590535); 429 bp in *Doleschallia melana*, Hamilton et al. 2020). The gene arrangement of the *M. ambigua* mitochondrial genome was identical to that observed in most lepidopteran genomes. Twelve of the identified PCGs had the typical ATN start codon, whereas *COI* had CGA, which is frequently found in members of the Lepidoptera. The A/T content of the whole mitogenome was 80.57%, well within the range found in Nymphalinae (79.39–80.90%; Alexiuk et al. 2020b; Payment et al. 2020b), and varied among the region/genes as follows: the A + T-rich region, 93.39%; *srRNA*, 85.37%; *lrRNA*, 84.92%; tRNAs, 81.13%; and PCGs, 79.22%. This pattern was detected in all Nymphalidae evaluated, except *Polygonia c-aureum*, in which the A/T content in *lrRNA* (84.76%) was slightly higher than that in *srRNA* (84.61%) (Shi et al. 2018).

Phylogenetic analyses placed *M. ambigua* in the tribe Melitaeini as a sister *Melitaea cinxia*, with this relationship having the highest nodal support in both ML (Figure 1) and BI analyses (data not shown). Additionally, the tribes Junoniini, Melitaeinii, and Nymphalini in Nymphalinae each formed a monophyletic group with the highest nodal support in both analyses. However, the tribe Kallimini was not supported as a monophyletic group, forming a sister group between *Doleschallia melana* in Kallimini and the monophyletic Melitaeinii in both analyses, although the nodal support was weak (bootstrap percentages, 51%, Figure 1; Bayesian posterior probabilities, 0.52, data not shown). Non-monophyletic Kallimini was also reported in previous phylogenetic studies, which used mitochondrial genomes (Alexiuk et al. 2020a; Aguila et al. 2021).

**Figure 1.**
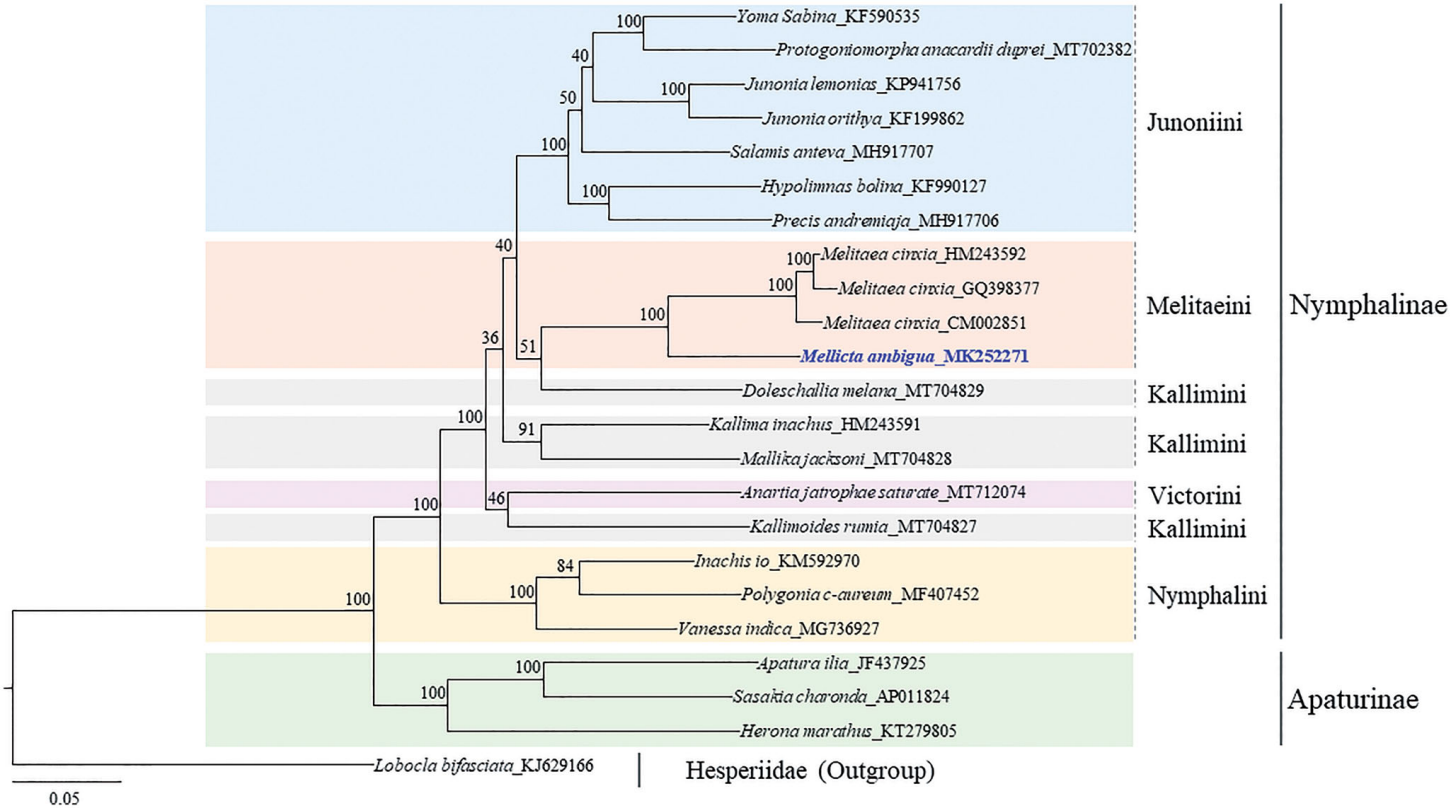
Phylogenetic tree of Nymphalidae. Maximum Likelihood (ML) method was used for the phylogenetic analysis based on concatenated sequences of 13 protein-coding genes (PCGs) and 2 rRNAs. The numbers at the nodes indicate bootstrap percentages of 1,000 pseudoreplicates by ML analysis. The scale bar indicates the number of substitutions per site. Hesperiidae (*Lobocla bifasciatus,* KJ629166, Kim et al. 2014) was used as the outgroup. Publication information is as follows: *Yoma Sabina*, KF590535 (Unpublished); *Protogoniomorpha anacardii duprei*, MT702382 (Lalonde and Marcus 2020); *Junonia lemonias*, KP941756, (McCullagh and Marcus 2015); *Junonia orithya*, KF199862 (Shi et al. 2015a); *Salamis anteva*, MH917707 (Lalonde and Marcus 2019b); *Hypolimnas bolina*, KF990127 (Shi et al. 2015b); *Precis andremiaja*, *MH917706* (Lalonde and Marcus 2019a); *Melitaea cinxia*, HM243592 (Xu et al. unpublished); *Melitaea cinxia,* GQ398377 (Hu et al. unpublished, GenBank accession number GQ398377); *Melitaea cinxia,* CM002851 (Ahola et al. 2014); *Mellicta ambigua*, MK252271 (This study); *Doleschallia melana*, MT704829 (Hamilton et al. 2020); *Kallima inachus*, HM243591 (Qin et al. 2012); *Mallika jacksoni*, MT704828 (Alexiuk et al. 2020b); *Anartia jatrophae saturate*, MT712074 (Payment et al. 2020a); *Kallimoides rumia*, MT704827 (Payment et al. 2020b); *Inachis io*, KM592970 (Timmermans et al. 2016); *Polygonia c-aureum*, MF407452 (Shi et al. 2018); *Vanessa indica*, MG736927 (Lu et al. 2018); *Apatura ilia*, JF437925 (Chen et al. 2012); *Sasakia charonda*, NC_014224 (Unpublished); and *Herona marathus,* KT279805 (Wang et al. 2016).

## Data Availability

The genome sequence data that support the findings of this study are openly available in GenBank of NCBI at https://www.ncbi.nlm.nih.gov/nuccore/MK252271.1
